# YAP-associated chromosomal instability and cholangiocarcinoma in mice

**DOI:** 10.18632/oncotarget.23638

**Published:** 2017-12-22

**Authors:** Sumera Ilyas, Samantha R. Fischbach, Steven F. Bronk, Petra Hirsova, Anuradha Krishnan, Renumathy Dhanasekaran, James B. Smadbeck, Rory L. Smoot, George Vasmatzis, Gregory J. Gores

**Affiliations:** ^1^ Division of Gastroenterology and Hepatology, Mayo Clinic, Rochester, 55905 MN, USA; ^2^ Institute of Clinical Biochemistry and Diagnostics, Charles University, Faculty of Medicine and University Hospital Hradec Kralove, Hradec Kralove 500 05, Czech Republic; ^3^ Department of Pharmacology, Charles University, Faculty of Medicine in Hradec Kralove, Hradec Kralove 500 03, Czech Republic; ^4^ Division of Gastroenterology and Hepatology, Stanford University, Stanford, 94304 CA, USA; ^5^ Department of Biomarker Discovery, Center for Individualized Medicine, Mayo Clinic, Rochester, 55905 MN, USA; ^6^ Department of Surgery, Mayo Clinic, Rochester, 55905 MN, USA

**Keywords:** chromosomal instability, FOXM1, mate-pair sequencing, SB cell lines

## Abstract

Deregulated Hippo pathway signaling is associated with aberrant activation of the downstream effector yes-associated protein (YAP), an emerging key oncogenic mediator in cholangiocarcinoma (CCA). In our prior work, we have demonstrated that biliary transduction of YAP along with Akt as a permissive factor induces CCA in mice. To further delineate the mechanisms associated with YAP-associated biliary oncogenesis, we have established seven malignant murine cell lines from our YAP-driven murine CCA model. These cells express the CCA markers SRY (Sex Determining Region Y)-Box 9 (SOX9), cytokeratin (CK)-7 and 19 but lack hepatocyte nuclear factor 4 alpha and alpha-smooth muscle actin, markers of hepatocellular carcinoma and cancer-associated fibroblasts, respectively. Notably, the murine CCA cells can be readily implanted into mouse livers with resultant orthotopic tumor formation. In this unique syngeneic orthotopic murine model, tumors exhibit histopathologic features resembling human CCA. We analyzed transcriptome data from YAP-associated parent CCA tumor nodules and identified a gene expression pattern associated with chromosomal instability, known as CIN25. Similarly, mate-pair sequencing of the murine CCA cells revealed chromosomal missegregation with gains and losses of several whole chromosomes demonstrating aneuploidy. Of the CIN25 genes, forkhead box M1 (Foxm1), a key cell cycle regulator, was the most significantly upregulated CIN25 gene product. Accordingly, small interfering RNA (siRNA)-mediated silencing of YAP as well as FOXM1 inhibition with thiostrepton induced CCA cell death. These preclinical data imply a role for YAP-mediated chromosomal instability in cholangiocarcinoma, and suggest FOXM1 inhibition as a therapeutic target for CCA.

## INTRODUCTION

Cholangiocarcinoma (CCA) is an aggressive hepatobiliary malignancy with markers of biliary epithelial cell differentiation and an increasing incidence [1]. Therapeutic options for advanced CCA are limited and 5-year survival remains less than 10% [2]. CCAs are characterized by extensive desmoplasia with a rich stroma of alpha-smooth muscle actin (α-SMA)-positive cancer-associated fibroblasts. The prominent tumor microenvironment and profound genetic heterogeneity of CCAs contribute to their therapeutic resistance [3]. Hence, an enhanced understanding of the molecular mechanisms underpinning CCA pathogenesis may herald new therapeutic targets. Preclinical CCA models are essential in elucidating the oncogenic signaling networks contributing to CCA pathogenesis and identification of novel therapeutic strategies.

The Hippo signaling pathway is an evolutionary conserved growth control pathway, and recently has emerged as a tumor suppressor pathway [4]. Deregulation of the Hippo pathway and consequent activation of its downstream effector, the transcriptional co-activator yes-associated protein (YAP), promotes carcinogenesis in a spectrum of malignancies including CCA [5–11]. Indeed, we have previously reported that biliary transduction with constitutively active human YAP (huYAP), YAPS127A, along with murine myristoylated-Akt (muAkt) as a permissive factor, promotes CCA development in mice [12]. We have also recently identified an oncogenic, feed-forward, autocrine YAP and fibroblast growth factor receptor (FGFR) pathway in CCA, further emphasizing that YAP is at the nexus of an oncogenic network [7].

Chromosomal instability (CIN), a phenomenon occurring with high rates of chromosome missegregation or loss/gains of whole chromosomes, is a fundamental feature of human solid organ malignancies; aneuploidy (presence of an abnormal number of chromosomes in a cell) is a consequence of CIN [13, 14]. The reduced mitotic fidelity associated with CIN promotes tumor genetic heterogeneity and enables acquisition of metastatic potential. Hence, the presence of CIN correlates with poor patient prognosis [13, 14]. The presence of CIN in patients can be identified using a CIN signature, consisting of a panel of 25 or 75 (CIN25, CIN75) genes whose expression is consistently correlated with total functional aneuploidy in several solid organ malignancies [13]. These CIN signatures were detected in hepatocellular carcinoma (HCC), and YAP in cooperation with forkhead box M1 (FOXM1) was identified as a driver of CIN gene expression in HCC [15]. YAP activation has also been reported to promote cell polyploidy via Akt signaling in HCC [16]. However, the relationship between YAP and CIN in CCA is unknown, especially whether downstream mediators of the events represent potential therapeutic targets.

Herein, we define several murine CCA cell lines derived from YAP-driven CCA tumors. These cells exhibit phenotypic features of human CCA, and can be readily implanted in mouse livers in an orthotopic fashion resulting in a unique syngeneic murine model of CCA. A CIN signature was detected in the YAP-driven murine tumors. Moreover, the murine cells exhibited chromosomal missegregation, implicating CIN as an oncogenic driver in CCA. Finally, inhibition of YAP or FOXM1 induced CCA cell death.

## RESULTS

### Murine tumor-derived cells express CCA phenotypic markers

**Figure 1:**
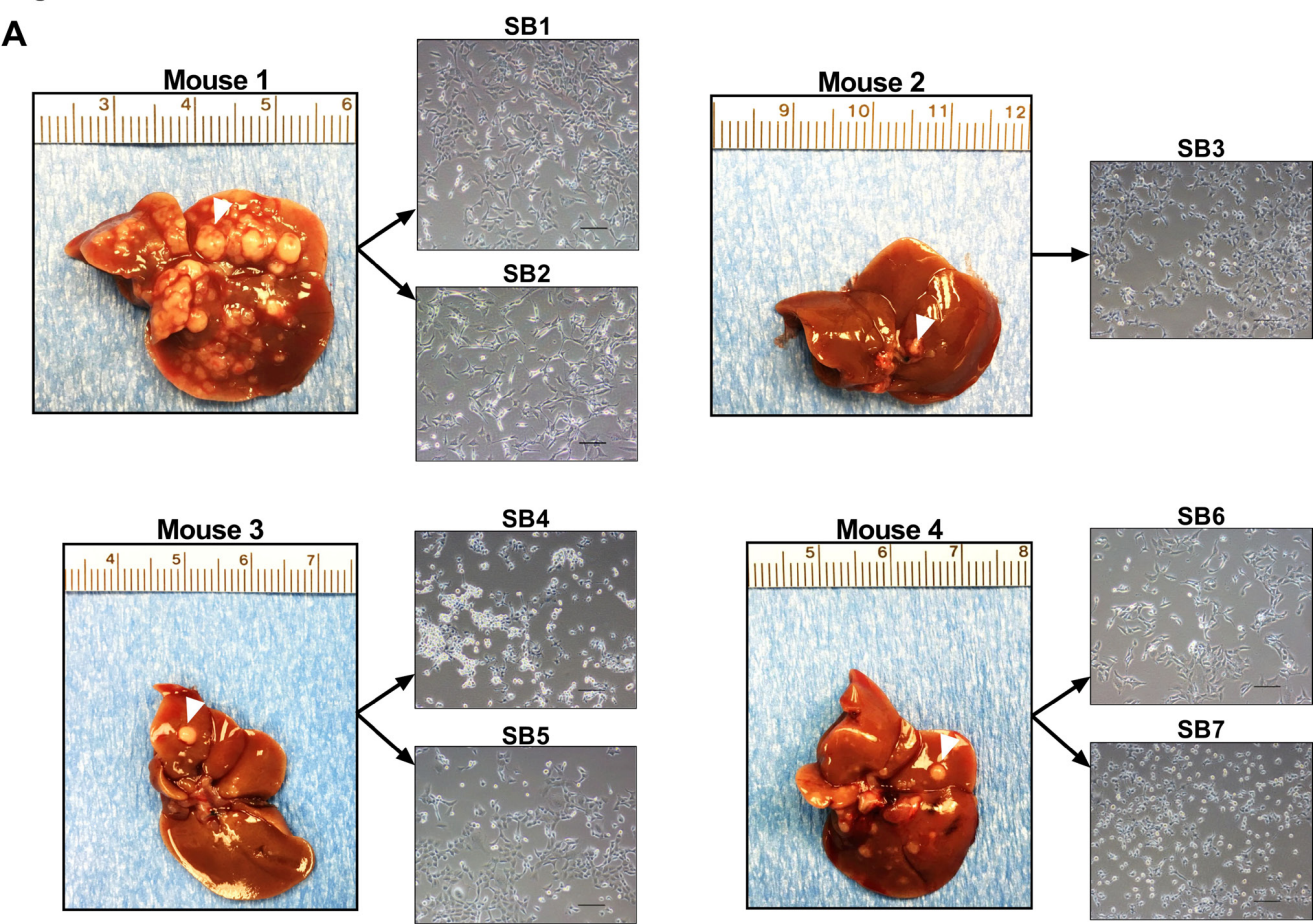

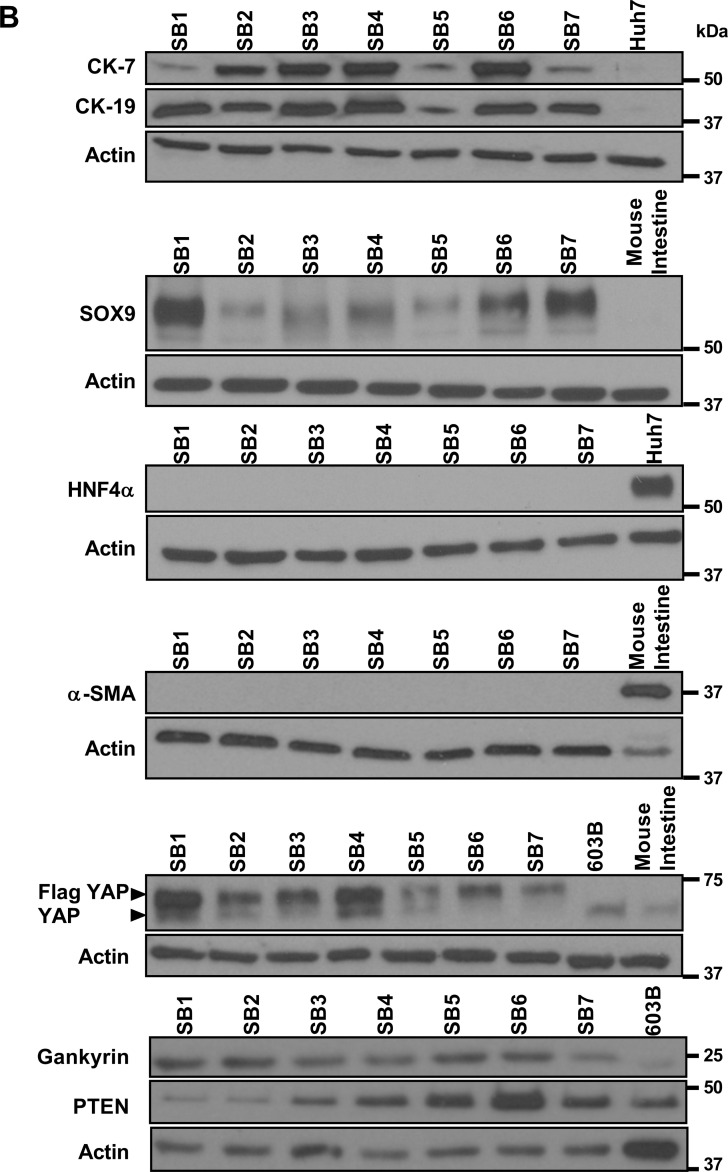
Murine cells derived from YAP-associated tumors have phenotypic features of human CCA (**A**) Liver appearances of mice 10 weeks after having undergone biliary transduction of SB+/–Akt+/–YAP coupled with subsequent systemic IL-33 administration (1 µg i.p. for 3 days) (left panels). Representative phase contrast microscopy images of murine cells derived from distinct tumor nodules (white arrow denotes representative nodule) (right panels). Original magnification 20×. Scale bars: 50 µm. (**B**) Whole cell lysates were prepared from SB1-7, Huh7, and mouse immortalized, nonmalignant cholangiocytes (603B). Lysates were also prepared from mouse intestine tissue. Lysates were subjected to immunoblot analysis of CK-7, CK-19, SOX9, HNF4α, α-SMA, FLAG-YAP, YAP, gankyrin, and PTEN. β-actin was used as a loading control.

**Figure 2:**
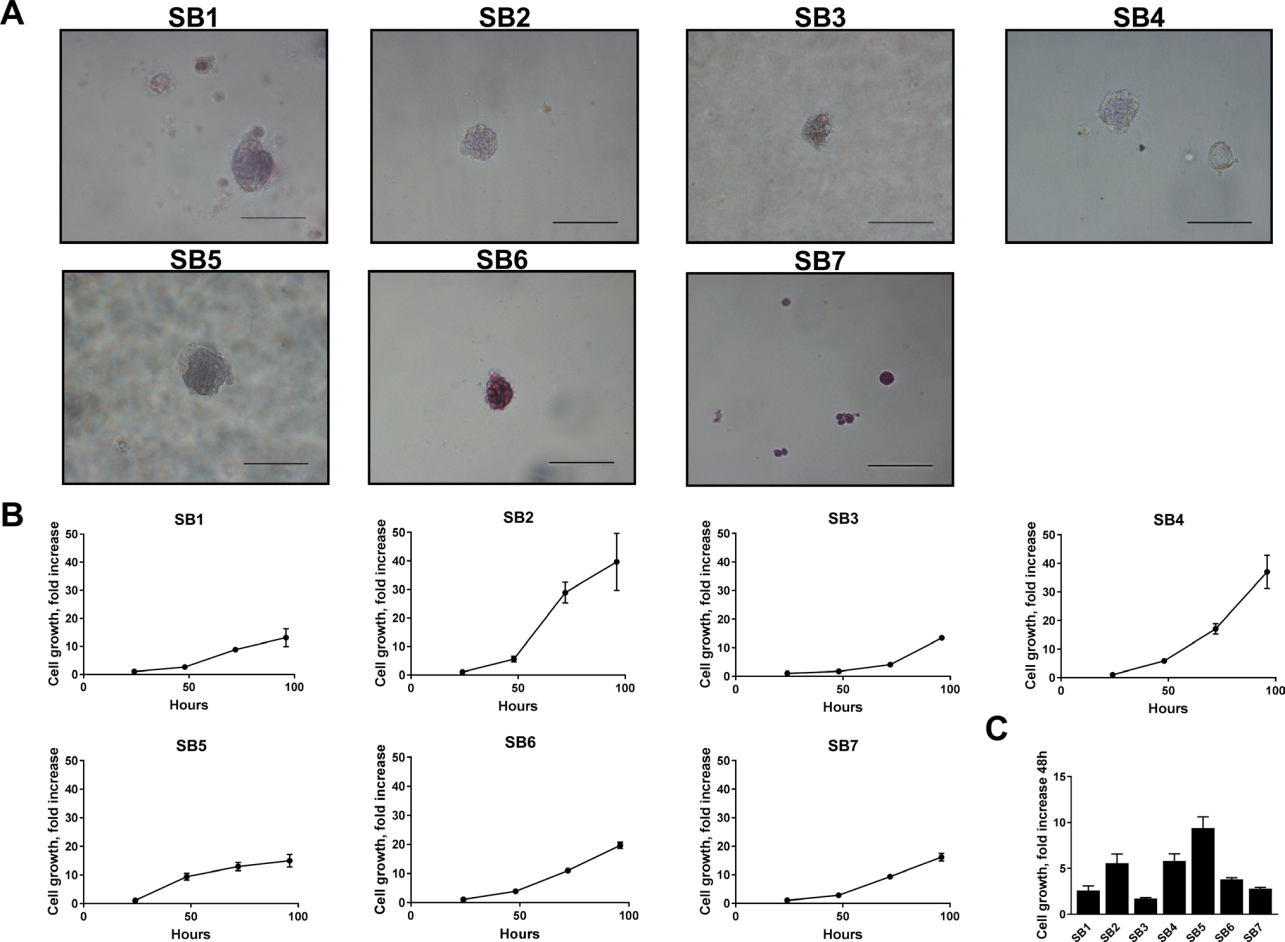
Murine cells derived from YAP-associated tumors have malignant features (**A**) SB1-7 cell lines were plated in soft agar and monitored until colony formation was detected. Representative images demonstrate colonies for each cell line. Original magnification 40×. Scale bars: 50 µm. (**B**) Growth curves of SB1-7 (1000 cells/well) 0, 24, 48, 72, and 96 hours after plating. (**C**) Comparative cell growth of SB1-7 48 hours after plating.

*S*even different primary cells (SB1-7) were derived from distinct tumor nodules harvested from C57BL/6 mice ten weeks after transposase-mediated transduction of constitutively active Akt and YAP in the biliary epithelium coupled with lobar obstruction and systemic interleukin-33 (IL-33) administration (Figure 1A). Conventional markers of CCA including cytokeratin (CK)-7 and 19 as well as SRY (Sex Determining Region Y)-Box 9 (SOX9) were abundantly expressed in SB1-7 cells, but not in the HCC cell line Huh7 or mouse intestine cells (Figure 1B). In contrast, hepatocyte nuclear factor 4 alpha (HNF4α), a marker of HCC was not expressed in the SB1-7 cells (Figure 1B). Similarly, SB1-7 cells also lacked expression of α-SMA, a marker of myofibroblasts/cancer-associated fibroblasts. Hence, the cells displayed features of CCA cells. Expression of FLAG-tagged huYAP was abundant in the SB1-7 cells and had not been lost during the transformation process (Figure 1B). Gankyrin, a component of the 19S proteosome, helps promote YAP-induced oncogenic activity in cholangiocarcinoma [11], and, therefore, we also assessed its expression in the SB1-7 cell lines. Indeed, consistent with its role in YAP-induced oncogenicity, gankyrin was abundantly expressed (Figure 1B). Although phosphatase and tensin homolog (PTEN) deficiency may promote gankyrin activity, PTEN was also abundant in the cell lines consistent with YAP’s pleiotropic oncogenic properties. Finally, these cells also displayed anchorage-independent colony formation in soft agar, a hallmark of malignant transformation (Figure 2A). Each cell line demonstrated progressive proliferation and relatively similar growth patterns (Figure 2B), albeit the SB5 cells exhibited the most rapid growth rate (Figure 2C). Taken together, these findings suggest that murine CCA cells SB1-7 are phenotypically similar to human CCA, display a malignant phenotype (anchorage-independent growth) and abundantly express FLAG-tagged huYAP.

### Implantation of SB cells results in orthotopic CCA tumor nodules

**Figure 3:**
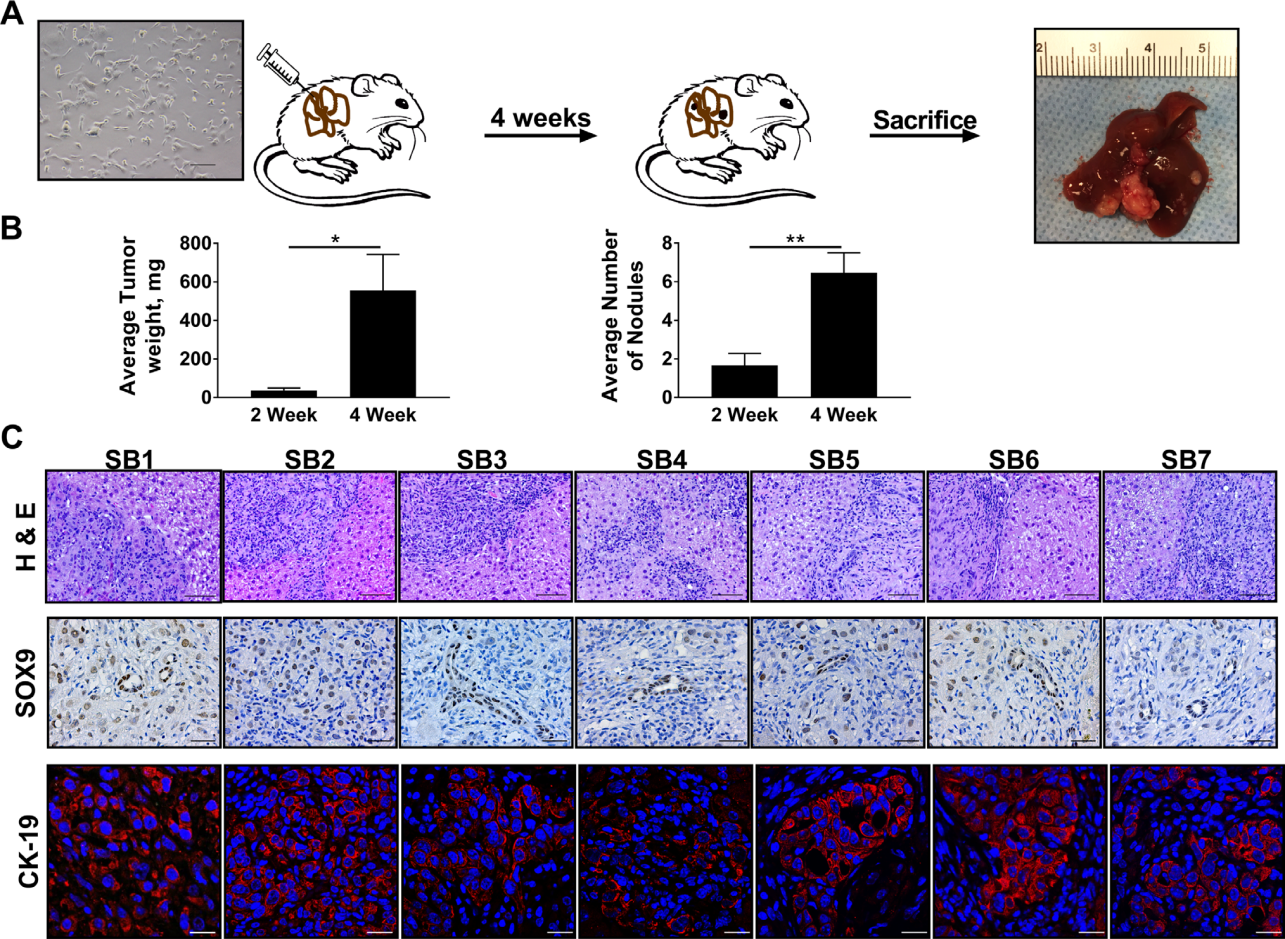
Implantation of SB cells facilitates biliary tumorigenesis in a syngeneic orthotopic murine model of CCA (**A**) Schematic diagram depicting implantation of SB cells into mouse livers with resultant tumor formation at four weeks. (**B**) Average tumor weights in milligrams (mg) in mouse livers 2 weeks (*n* = 6) and 4 weeks (*n* = 13) following orthotopic implantation of SB1 cells (left panel). Average number of nodules in mice livers 2 weeks (*n* = 6) and 4 weeks (*n* = 13) following orthotopic implantation of SB1 cells (right panel). ^*^p < 0.05; ^**^p < 0.01. (**C**) Representative photomicrographs of hematoxylin and eosin-stained tumor sections and adjacent livers from mice having undergone orthotopic implantation of SB cells (upper panels). Original magnification 20×. Scale bar: 50 µm. Immunohistochemistry was used to detect SOX9 expression in tumors derived from implantation of SB1-7; representative images of random fields within the tumor tissue specimens from the same animal are shown (middle panels). Original magnification 40×. Scale bar: 20 µm. Immunofluorescence was used to detect CK-19 expression in tumors derived from implantation of SB1-7; representative images of random fields within the tumor tissue specimens from the same animal are shown (bottom panels). Original magnification 63×. Scale bar: 20 µm.

Next, we set out to determine whether orthotopic implantation of SB1-7 murine CCA cells into mice results in tumor formation in male C57BL/6 mice; a sine qua non of malignancy. Four weeks after implantation of SB cells, tumor development was noted in all mouse livers (Figure 3A). A significant increase in tumor weight and number of nodules was noted when mice were sacrificed 4 weeks following orthotopic SB cell implantation compared to 2 weeks following implantation (Figure 3B). Histologically, murine tumors resulting from implantation of any of the SB cells distinctly resembled human CCAs with the presence of classic morphologic features including desmoplasia and malignant gland hyperplasia (Figure 3C). Similar to human CCA specimens, these murine tumors had enhanced expression of SOX9 and CK-19 compared to adjacent non-tumor liver. Hence, SB cells display key features of malignancy including tumor formation following implantation into a syngeneic background. The ability to orthotopically implant the cells into a syngeneic background will permit investigation of the tumor microenvironment in CCA, and can be exploited for therapeutic studies.

### SB cell lines display chromosomal instability

**Figure 4:**
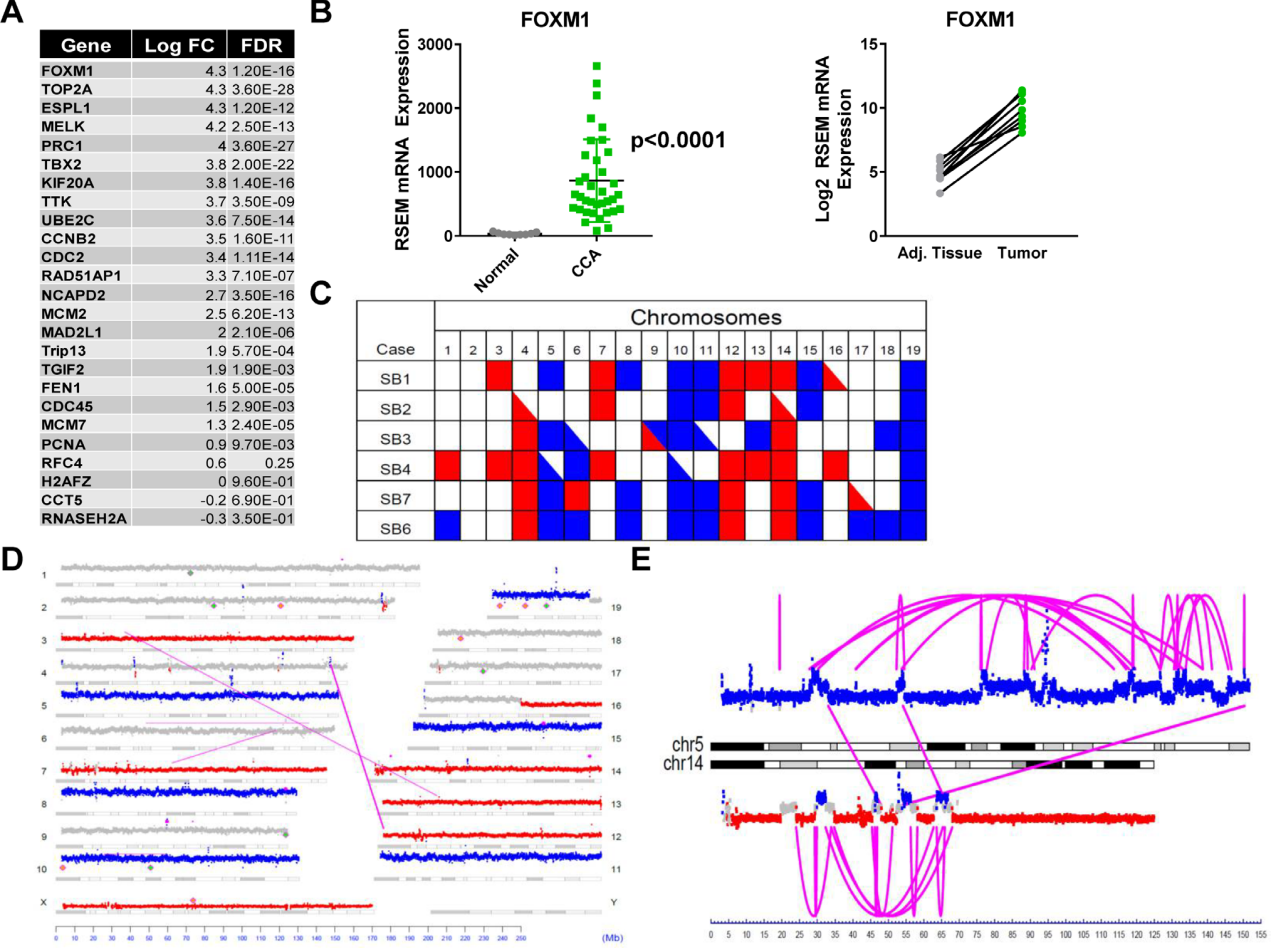
YAP-associated murine tumors and cells exhibit chromosomal instability (CIN) (**A**) Expression levels of CIN genes (CIN25 signature) in Akt-YAP mouse tumors compared to corresponding adjacent liver. (**B**) FOXM1 expression in CCA tumor tissue compared to adjacent liver tissue using RNA sequencing data from TCGA-Chol patient cohort. (*p* < 0.0001; mean FC 25) (left panel). Comparison of FOXM1 gene expression in paired tumor-adjacent tissue (adj.) samples (right panel) from the TCGA-Chol patient cohort. (**C**) Copy number variation heat map using MPseq analysis of SB1-4, SB6-7. Copy number variation results are summarized for each chromosome in each sample. A red square indicates that all of a chromosome has been lost (monosomy). A blue square indicates that all of a chromosome has been gained (trisomy). Red and blue triangles indicate that a large copy number loss or gain is present but that it does not cover the entire chromosome. (**D**) Genome plot for cell line SB1. The genome plot presents the full mate-pair analysis for cell line SB1. Autosomes are numbered 1 to 19 and allosomes are labeled X and Y. Each chromosome panel is represented by its (a) G banding phenotype, (b) dots denoting sequencing coverage and (c) diamonds representing integration sites of huYAP (*orange diamonds*) or muAkt (*green diamonds*). Each dot represents 30000 bases. Regions calculated to have normal copy number level are colored gray. Regions of data are colored blue for gain of genetic material or red for loss of genetic material. Inter- and intra-chromosomal breakpoint junctions are presented as magenta lines linking two breakpoint positions. (**E**) A detailed view of a chromoplexy rearrangement in cell line SB6. This chromoplexy rearrangement involving chromosomes 5 and 14 is characterized by a large number of intra- and interchromosomal breakpoint junctions shown by magenta arcs and lines connecting two breakpoint positions. Regions colored in gray indicate normal 2N copy number. Copy number variants resulting from this rearrangement are colored red to indicate genetic loss and blue to indicate genetic gain. FC, fold change; FDR, false discovery rate.

CIN is a uniform characteristic of solid organ malignancies including CCA, and is associated with tumor metastasis and poor clinical outcome [13]. RNA sequencing analysis of the parent tumors from which SB1-7 cells were derived, was utilized to assess for the presence of a CIN signature, a gene expression pattern associated with chromosomal instability. Expression of 25 genes associated with functional aneuploidy (CIN25 signature) [13] including FOXM1 was upregulated in the mouse tumors compared to adjacent liver (Figure 4A). YAP induces FOXM1 to promote CIN in HCC [15], and hence the upregulation of FOXM1 is mechanistically relevant. Accordingly, we observed marked upregulation of FOXM1 gene expression in human CCA tumors compared to adjacent tissue in the cancer genome atlas cholangiocarcinoma cohort (TCGA-Chol) (Figure 4B). We next employed mate-pair sequencing (MPseq) to assess whether CIN was present in SB1-7 cells. MPseq, an innovative sequencing technology, is a comprehensive yet cost-effective method to detect the presence of large genomic chromosomal rearrangements such as chromosomal amplifications and deletions [17]. MPseq analysis of SB1-4, SB6-7 demonstrated gains and losses of multiple chromosomes across the cell lines consistent with CIN and aneuploidy (Figures 4C, 4D, Supplementary Figures 1–5). MPseq of SB5 could not be performed due to repeated suboptimal quality control. Loss of genetic material from chromosomes 4, 12, and 14 was detected in the majority of the SB cell lines (Figure 4C, 4D, Supplementary Figures 1–5). SB1 and SB4 also had losses in chromosomes 3 and 13 (Figure 4D, Supplementary Figure 3). The majority of SB cells exhibited gains in chromosomes 5, 8, 10, 11, 15, and 19 (Figure 4C, 4D, Supplementary Figures 1–5). Gains in chromosome 6 were detected in SB3, SB4, and SB6 (Supplementary Figures 2–4) whereas gains in chromosome 8 were detected in SB1, SB6, and SB7 (Figure 4D, Supplementary Figures 4–5). Chromoplexy, the phenomenon of complex weaving genomic rearrangements occurring in the genomes of cancer cells, was also detected in the SB cells [18, 19]. For instance, a chromoplexy rearrangement characterized by a large number of intra-and interchromosomal breakpoint junctions involving chromosomes 5 and 14 was observed in the SB6 cell line (Figure 4E). Collectively, these data suggest that CIN contributes to YAP-mediated tumorigenesis in murine CCA tumors and cells.

### YAP siRNA-directed knockdown or FOXM1 pharmacologic inhibition induces murine CCA cell death

**Figure 5:**
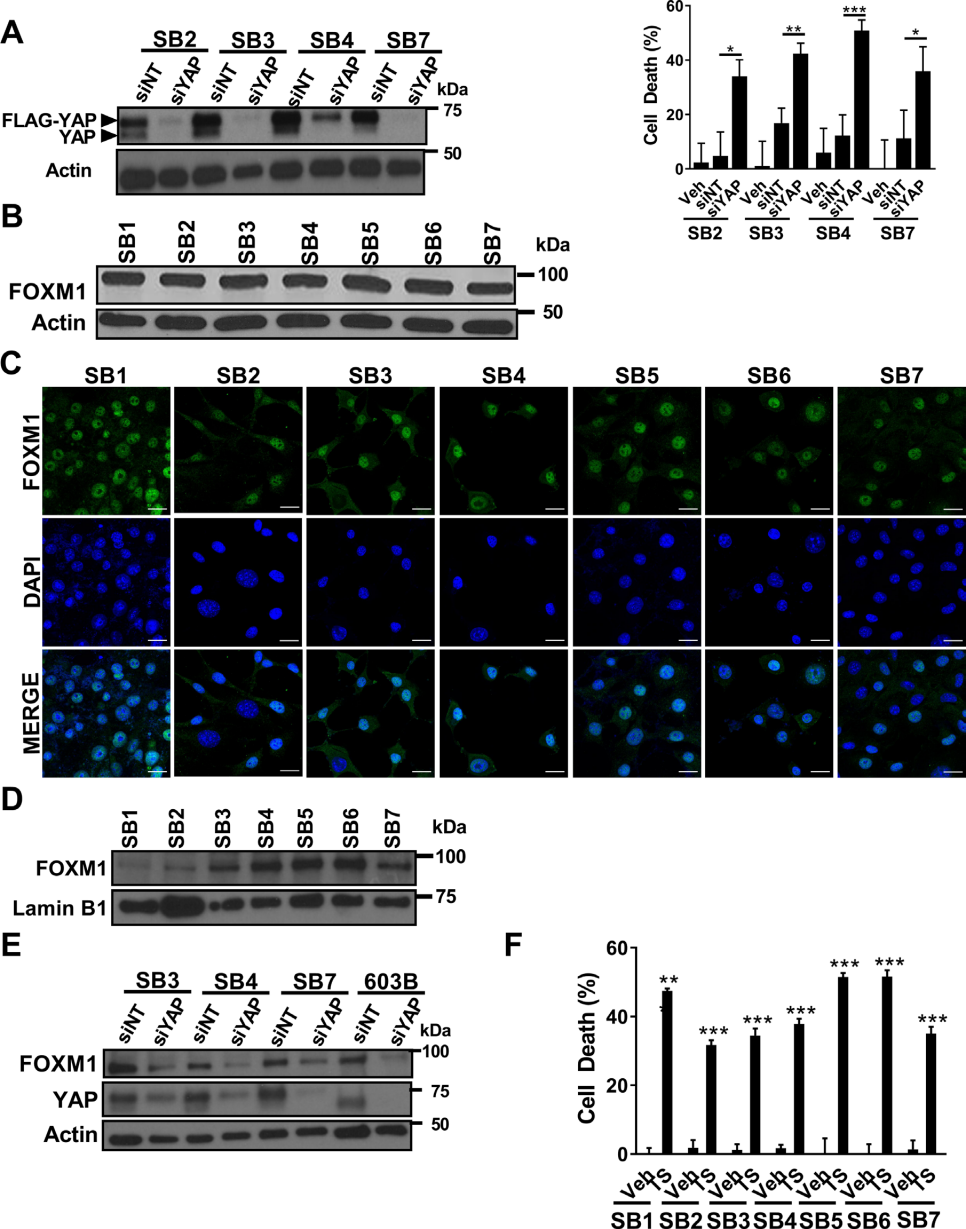
YAP siRNA-directed knockdown or FOXM1 pharmacologic inhibition induces murine CCA cell death (**A**) Immunoblot analysis of FLAG-YAP and YAP in SB2, SB3, SB4, SB7 cells 72 h after transfection with siRNA targeting YAP (siYAP) or non-targeting siRNA (siNT) as a control (left panel). β-actin was used as a loading control. Cell death rate was evaluated 72 h following transfection of SB2, SB3, SB4, and SB7 cells with siYAP or siNT and compared to vehicle (veh) treated cells by CellTiter-Blue assay (right panel). ^*^p < 0.05; ^**^p < 0.01; ^***^p < 0.001. (**B**) Whole cell lysates from SB1-7 were subjected to immunoblot analysis of FOXM1. β-actin was used as a loading control. (**C**) Immunofluorescence images of FOXM1-positive nuclei in SB1-7. Scale bars: 20 µm. (**D**) Immunoblot analysis of FOXM1 in SB1-7 nuclear extracts. Lamin B1 was used as a loading control. (**E**) Immunoblot analysis of FOXM1 and YAP in SB3, SB4, SB7, and 603B cells 72 h after transfection with siRNA targeting YAP (siYAP) or non-targeting siRNA (siNT) as a control (left panel). β-actin was used as a loading control. (**F**) SB1-7 cells were treated with vehicle (veh) or thiostrepton (TS, 1 µM) for 24 h. Cell death rate was subsequently evaluated by CellTiter-Blue assay. ^**^p < 0.01; ^***^p < 0.001

**N**ext, we set out to determine whether SB cells were YAP or Akt dependent. Small interfering RNA (siRNA)-mediated knockdown of YAP in these cell lines induced significant cell death in SB2-4 and SB7 (Figure 5A). Transfection reagent toxicity precluded evaluation of cell death in SB1, 5, and 6. In contrast, all seven murine cell lines were resistant to cell death when incubated with the Akt inhibitor MK2206 (data not shown), despite efficient inhibition of Akt activating phosphorylation by the drug (Supplementary Figure 6). FOXM1 was upregulated in the parent CCA tumors; to confirm that FOXM1 was also expressed in the cell lines we performed immunoblot analysis and immunocytochemistry. Indeed, we identified FOXM1, a transcription factor, protein expression (Figure 5B) and nuclear localization by immunofluorescence and immunoblot analysis (Figure 5C and 5D) in all examined cell lines. Consistent with prior reports [15], FOXM1 expression was dependent upon YAP expression as siRNA targeted knockdown of YAP also reduced FOXM1 protein abundance (Figure 5E). Given that YAP induces FOXM1 in our cell lines, and that FOXM1 has been proposed to be a therapeutic target in breast cancer [20], we next sought to assess whether FOXM1 inhibition induces CCA cell death. Indeed, treatment of SB1-7 cells with thiostrepton, a FOXM1 inhibitor [21], resulted in significant cell death in all the murine cell lines (Figure 5F). These observations are most compatible with YAP-dependence of these murine CCA cell lines, and suggest that FOXM1 inhibition may be therapeutic in CCA.

## DISCUSSION

This study describes several unique murine CCA cell lines and a new syngeneic orthotopic murine model of CCA. These data indicate that: (i) murine cells derived from Akt-YAP driven tumors have phenotypic features of human CCA; (ii) implantation of murine CCA cells results in development of orthotopic tumors which are morphologically and phenotypically similar to human CCA; (iii) the cell lines exhibit aneuploidy and chromosomal instability with upregulation of FOXM1; (iv) the cell lines are YAP-dependent and FOXM1 pharmacologic inhibition causes CCA cell death. These findings are discussed in detail below.

The Hippo signaling pathway is a highly conserved growth control pathway which regulates a variety of genes implicated in diverse cellular processes such as proliferation, differentiation, and survival [4]. The Hippo pathway kinase module restrains YAP signaling under basal conditions; inactivation of the Hippo pathway leads to YAP nuclear translocation with consequent activation of pro-proliferative and pro-survival genes. Accordingly, Hippo signaling deregulation and YAP overexpression has been implicated in a multitude of malignancies including CCA [5–8]. We have previously demonstrated that biliary instillation of constitutively active human YAPS127A with mouse myristoylated Akt as a permissive factor fosters intrahepatic CCA formation in mice, implicating YAP as a critical oncogene in biliary carcinogenesis [12]. We have now derived seven murine CCA cell lines from distinct Akt-YAP tumor nodules. These cell lines exhibit characteristic features of malignancy such as anchorage-independent growth. Moreover, all seven cell lines morphologically resemble human CCA cells displaying several CCA phenotypic characteristics such as SOX9 and CK-7/19 expression. Interestingly, these cell lines remains YAP-dependent as inhibition of YAP expression by targeted siRNA causes cell death.

We have also demonstrated that all seven cell lines can be readily implanted into mouse livers with consequent tumor formation and generation of a syngeneic orthotopic model of CCA. Similar to the YAP-driven genetic murine model, these orthotopic tumors have histopathologic and phenotypic features of human CCA. This model could have significant utility in investigating oncogenic signaling pathways in CCAs as it allows for manipulation of the murine cells prior to transplantation. For instance, transfection of the cells with inducible genes or inhibition constructs may elucidate the impact of various oncogenes in CCA progression and survival. Moreover, this model can be exploited to examine the immunologic response and stroma formation in CCA as well as investigate new therapies for CCA.

Aberrant YAP activity has also recently been implicated as a driver of CIN and aneuploidy [15]. YAP induces and interacts with FOXM1, a master regulator of cell-cycle control, by forming a multimeric, transcriptionally active complex comprising YAP, FOXM1, as well as TEA domain transcription factor 4 (TEAD4), a YAP binding partner [15]. This YAP/TEAD4/FOXM1 complex drives CIN gene expression and induces aneuploidy [14]. FOXM1 is a transcription factor with an essential role in cell-cycle specific gene regulation. FOXM1 is essential for transcription of mitotic regulatory genes, cell cycle progression, and maintenance of chromosomal stability [22, 23]. Hence, FOXM1 expression is diminished in quiescent cells but is upregulated at the onset of the S-phase of the cell cycle in actively dividing normal cells (e.g. in embryonic tissues) [23–25]. However, FOXM1 overexpression is also observed in a spectrum of malignancies and is associated with transcriptional activation of its targets as well as increased proliferation of malignant cells [26–28]. Accordingly, FOXM1 is a component of the CIN25/75 gene signatures, and regulates several CIN genes [13]. We detected the CIN25 signature in YAP-associated murine tumors. Interestingly, our data indicated that FOXM1 was the most significantly upregulated of the CIN25 genes, suggesting a key oncogenic role of FOXM1 in these tumors. Consistent with this, marked FOXM1 upregulation in human CCA was confirmed in the TCGA CCA cohort. Interestingly, inhibition of FOXM1 with thiostrepton induced cell death in all seven murine CCA cell lines, implying it is a potential therapeutic target for CCA. This observation is consistent with synthetic lethality for FOXM1 cell dependence in the context of YAP-driven oncogenic transformation.

We employed MPseq analysis, a sophisticated yet cost-effective sequencing technology, to examine large genomic rearrangements such as gains and losses of chromosomes in the SB cell lines. MPseq detects the presence of aneuploidy by uncovering structural variants throughout the entire genome. Utilizing MPseq, we identified chromosomal instability, as signified by the presence of chromosomal missegregation with gains and losses of several whole chromosomes, as a prominent feature in the SB cell lines. To our knowledge, this is the first utilization of MPseq in a mouse model of cancer.

In summary, we have developed several murine CCA cell lines and a unique syngeneic orthotopic murine model of CCA. Our study augments and supports the notion that YAP is a critical mediator in biliary oncogenesis. Moreover, these findings suggest that YAP promotes CIN in cholangiocarcinoma. Our syngeneic model is a potentially valuable tool which can be employed to enhance our understanding of CCA pathogenesis and therapeutics. Finally, loss of cell viability with FOXM1 inhibition suggests that FOXM1 may be a potential therapeutic target for CCA. Further preclinical studies are warranted to confirm FOXM1 inhibition as a viable therapeutic approach in CCA.

## MATERIALS AND METHODS

### Cell culture and reagents

The mouse cholangiocarcinoma cell lines SB1-SB7 were cultured in Dulbecco’s Modified Eagle Medium (DMEM) supplemented with 10% fetal bovine serum (FBS), 0.2% primocin, and 0.01% insulin under standard conditions. The murine nonmalignant, immortalized cholangiocyte cell line 603B [29] was cultured in Eagle’s minimum essential medium supplemented with 10% FBS, penicillin (1,000 U/mL), and streptomycin (100 µg/mL). The human HCC cells, Huh7, were cultured in DMEM containing glucose (4.5 g/L), penicillin (100 U/mL), streptomycin (100 µg/mL) and 10% FBS (Gibco, Carlsbad, CA, USA) under standard conditions. The FOXM1 inhibitor thiostrepton [21] (Sigma, St. Louis, MO, USA) was dissolved in dimethyl sulfoxide (DMSO) and added to cells in a final concentration of 1 µM for 24 hours. DMSO was used as a control.

### Establishment of primary murine tumor cell cultures

C57BL/6 mice underwent biliary transduction of sleeping beauty transposon-transposase, muAkt and huYAP coupled with unilobar bile duct ligation and systemic IL-33 administration, as previously described [12]. Note that the human YAP is FLAG-tagged and hence can be distinguished from endogenous mouse YAP. Mice were sacrificed 10 weeks after biliary oncogene transduction and tumor tissue was harvested. Murine tumors were dissociated using the mouse Tumor Dissociation Kit (130-096-730; Miltenyi Biotech, Auburn, CA, USA) and the gentleMACS Octo Dissociator with heaters (Miltenyi Biotech, Auburn, CA, USA) according to manufacturer’s protocol. Dissociated tumor material from each separate nodule was passed through a sterile 60-µM mesh filter and plated in 6-well plates containing standard media. Following attachment of cellular material, media was changed every 3–4 days. Attached cells were trypsinized and re-plated when near-confluence. Surviving cells established colonies. Seven cell lines were established and termed SB1-7.

### Anchorage-independent cell growth in soft agar

A base layer of soft agar was formed in 6-well plates using 0.9% of a low melting agarose (SeaPlaque Agarose; Lonza, Basel, Switzerland) in 1 mL of DMEM, and allowed to solidify. Next, 1 mL of 0.9% agarose in DMEM containing 2500 cells was layered atop the base agarose layer and allowed to solidify. Subsequently, 1 mL of DMEM was added to each well and plates were incubated at 37°C until colony establishment was noted in 10–20 days. Colonies were then fixed in PBS with 4% formaldehyde and stained with 0.005% crystal violet for 1 hour. Staining media was removed and colonies were imaged using a phase contrast microscope (Nikon Eclipse TE300).

### Cell growth curves

SB1-7 cells were plated in 96-well plates (1000 cells/per well) in DMEM. Cells were counted at 24, 48, 72 and 96 hours using Celigo Imaging Cytometer (Nexcelom Bioscience, Lawrence, MA, USA).

### Immunoblot analysis

Whole cell lysates or nuclear proteins extracted using a nuclear extraction kit (Thermo Fisher Scientific Inc.) were prepared as detailed previously [30]. Proteins were resolved by SDS-PAGE and transferred to nitrocellulose membranes. The following primary antibodies were used for immunoblot analysis: α-SMA (ab5694), FOXM1 (ab180710), SOX9 (ab5535) from Abcam (Cambridge, MA, USA); gankyrin (12985S), Lamin B1 (13435S), phospho-AKT (9271S), PTEN (9188S) from Cell Signaling Technology (Danvers, MA); AKT (07-416) from Millipore (Burlington, MA); β-Actin (sc-1615), CK-7 (sc-70936), CK-19 (sc-33119), HNF4α (sc-6556), YAP (sc-101199) from Santa Cruz Biotechnology (Santa Cruz, CA). Membranes were blotted with primary antibody overnight at 4°C at a dilution of 1:1000 for all primary antibodies. Horseradish peroxidase–conjugated secondary antibodies for rabbit (HAF008; 1:3000) and goat (SC-2020; 1:3000) were obtained from R&D Systems (Minneapolis, MN) and Santa Cruz Biotechnology (Santa Cruz, CA), respectively. Proteins were visualized with enhanced chemiluminescence reagents (ECL/ECL Plus, Amersham GE) and Kodak X-OMAT film.

### RNA sequencing from mouse CCA

mRNA was isolated from mouse tumor nodules and adjacent liver using the RNeasy Plus mini kit (Qiagen, Germantown, MD, USA). Sequencing of RNA was conducted by the Mayo Medical Genomics Facility. RNA libraries were prepared according to the manufacturer’s instructions for the TruSeq RNA Sample Prep Kit v2 (Illumina, San Diego, CA, USA). The liquid handling Eppendorf (Hamburg, GER) EpMotion 5075 robot was employed for TruSeq library construction. All AMPure bead clean up, mRNA isolation, end repair and A-tailing reactions was completed on the 5075 robot. Reverse transcription and adaptor ligation was performed manually. Briefly, poly-A mRNA was purified from total RNA using oligo dT magnetic beads. The purified mRNA was fragmented at 95°C for 8 minutes, eluted from the beads and primed for first strand cDNA synthesis. The RNA fragments were then copied into first strand cDNA using SuperScript III reverse transcriptase and random primers (Invitrogen, Carlsbad, CA, USA). Next, second strand cDNA synthesis was performed using DNA polymerase I and RNase H. The double-stranded cDNA was purified using a single AMPure XP bead (Agencourt, Danvers, MA, USA) clean-up step. The cDNA ends were repaired and phosphorylated using Klenow, T4 polymerase, and T4 polynucleotide kinase followed by a single AMPure XP bead clean-up. The blunt-ended cDNAs were modified to include a single 3′ adenylate (A) residue using Klenow exo- (3′ to 5′ exo minus). Paired-end DNA adaptors (Illumina) with a single “T” base overhang at the 3′ end were immediately ligated to the ‘A tailed’ cDNA population. Unique indexes, included in the standard TruSeq Kits (12-Set A and 12-Set B) were incorporated at the adaptor ligation step for multiplex sample loading on the flow cells. The resulting constructs were purified by 2 consecutive AMPure XP bead clean-up steps. The adapter-modified DNA fragments were enriched by 12 cycles of PCR using primers included in the Illumina Sample Prep Kit. The concentration and size distribution of the libraries were determined on an Agilent Bioanalyzer DNA 1000 chip (Santa Clara, CA, USA). A final quantification, using Qubit fluorometry (Invitrogen, Carlsbad, CA, USA), was done to confirm sample concentration. Libraries were loaded onto paired end flow cells at concentrations of 8–10 pM to generate cluster densities of 700,000/mm^2^ following Illumina’s standard protocol using the Illumina cBot and cBot Paired End Cluster Kit version 3. The flow cells were sequenced as 51 X 2 paired end reads on an Illumina HiSeq 2000 using TruSeq SBS Sequencing Kit version 3 and HCS v2.0.12 data collection software. Base-calling was performed using Illumina’s RTA version 1.17.21.3.

### RNA sequencing bioinformatics analysis workflow

Bioinformatics analysis was performed by the Mayo Bioinformatics Core Facility to determine differential expression of mouse tumor tissue and adjacent liver tissue. The processing of the mRNA data was performed using MAP-RSeq (v1.2.1.3) [31]. MAP-RSeq consists of the following steps: alignment, quality control, obtaining genomic features per sample and finally summarizing the data across samples. The pipeline provides detailed quality control data across genes using the RSeQC (v2.3.2) software [32]. Paired-end reads are aligned by TopHat (v2.0.6) [33] against the mm10 genome build using the bowtie1aligner option [34]. Gene counts were generated using HTSeq (v0.5.3p9) software and the gene annotation files were obtained from Illumina. Differential expression analysis comparing mouse tumor tissue vs. adjacent liver computed using the edgeR package (v2.6.2) [35]. This transcriptome data was used to compare the expression of the CIN25 genes (the top 25 genes known to be associated with chromosomal instability) [13] in mouse tumor tissue vs. adjacent liver.

### TCGA analysis

mRNA expression data generated by TCGA from human cholangiocarcinoma specimens (36 tumors; 9 surrounding normal tissues) was analyzed. The file containing level 3 normalized RSEM (RNA-Seq by Expectation Maximization) data from the Firehose run of the Broad Genome Data Analysis Center (Broad Firehose Data Accessed on 9/23/2017. https://doi.org/10.7908/C11G0KM9) was downloaded. Nonparametric Mann–Whitney test was used to perform differential expression analysis.

### Mate-pair sequencing

MPseq entails generation of long-insert paired-end DNA libraries which can be utilized for identification of large complex genomic rearrangements [17]. The cell lines SB1-7 were grown to 70–90% confluency. Genomic DNA (gDNA) was isolated per standard protocol and quality assessed by Agilent Bioanalyzer 2100 and quantity on a_Qubit Fluorometer using the Qubit dsDNA high sensitivity assay kit (Thermo Fisher Scientific, Q32854). Cell line SB5 repeatedly failed to pass the QC check and was omitted from further processing and analysis. For all other cell lines, sequencing libraries were prepared using the Illumina Nextera Mate-pair library prep kit (Illumina, FC-132-1001) from 1 µg of gDNA. Sequencing (paired end, 150 bp) was performed on an Illumina HiSeq 4000 by the Sequencing Core of Mayo Clinic’s Medical Genome Facility, Rochester, MN, USA.

### MPseq analysis

Mate-pair sequencing data was mapped to the GRCm38 reference genome modified to include the huYAP and muAkt sequences used in this study. This mapping was performed using our previously published binary mapping algorithm, BIMA [17]. The mapped fragments were run through a suite of bioinformatics algorithms called SVAtools. SVAtools includes algorithms for detecting breakpoint junctions and copy number variations in a given sample. The breakpoint junction detection algorithm clustered the mapped DNA fragments to find sets of read pairs that map to two distant locations in the reference genome (defined as locations mapped to different chromosomes or > 30Kb apart on the same chromosome). These clusters were classified as deletions, inversion, gains, and interchromosomal translocations. Additionally, as the transfected huYap and muAkt sequences were included in the reference genome, the integration sites for these genes were also detected. The copy number variation (CNV) detection algorithm was performed on whole-genome read depth data binned in 1Kb windows to detect gains and losses in genetic material. The read depth data was normalized using a set of previously sequenced normal samples and GC content in order to reduce false positives. Often the detected CNVs were the result of chromosomal rearrangements and were supported by breakpoint junctions, which were used to increase the resolution of the CNV calls. Additionally, the algorithm used a statistical sliding window segmentation algorithm in order to detect CNVs that are not expected to have junction support, like partial- and whole-chromosome aneuploidy. The detected CNV regions were classified as gain, normal, or loss based on the estimated 2N copy number level. The final result of this MPseq analysis pipeline was a full list of large structural variants in a sample. This included any breakpoint junctions and copy number variants that take part in chromosomal rearrangements, partial and whole-chromosome aneuploidy, and integration sites of the transfected huYap and muAkt sequences.

### Orthotopic, syngeneic mouse model of CCA

All animal experiments were performed in accordance with a protocol approved by the Mayo Clinic Institutional Animal Care and Use Committee. Murine CCA cells (SB1-7) were harvested and washed in DMEM. Male C57BL/6 mice from Jackson Labs were anesthetized using 1.5–3% isoflurane. Under deep anesthesia, the abdominal cavity was opened by a 1 cm incision below the xiphoid process. A sterile cotton tipped applicator was used to expose the superolateral aspect of the medial lobe of the liver. Using a 27-gauge needle, 40 µL of standard media containing 1 × 10^6^ cells was injected into the lateral aspect of the medial lobe. Cotton tipped applicator was held over the injection site to prevent cell leakage and blood loss. Subsequently, the abdominal wall and skin were closed in separate layers with absorbable chromic 3–0 gut suture material.

### Immunohistochemistry in mouse liver specimens

Liver tissue from euthanized mice was fixed in 4% paraformaldehyde for 48 hours, embedded in paraffin, and sectioned into 3–5 μm slices. Paraformaldehyde-fixed, paraffin-embedded mouse tumor and adjacent liver sections were deparaffinized, hydrated and incubated with primary antibody overnight at 4°C. Sections were stained with antibody for SOX9 (ab5535; 1:2000) from Millipore (Billerica, MA, USA). Primary antibody was detected with HRP-conjugated secondary antibody and diaminobenzidine (Dako, Carpentaria, CA, USA). Liver tissue sections were counterstained with hematoxylin.

### Immunofluorescence and immunocytochemistry

Paraformaldehyde-fixed, paraffin-embedded liver tissue sections were deparaffinized, hydrated, and permeabilized with Triton-X-100. Cells were seeded on Chamber Slide^™^ (Thermo Fisher Scientific) at 50% confluence. Following their respective treatments, the cells were fixed with 4% paraformaldehyde. After permeabilization using Triton-X-100, slides were then blocked for one hour at room temperature with 5% bovine serum albumin (BSA) and 0.1% glycine PBS before being incubated overnight with primary antibody at 4°C. Antibodies were diluted in phosphate buffered saline with 5% BSA at the following dilutions: CK-19 (sc-33119; 1:50) and FOXM1 (sc-376471; 1:100) from Santa Cruz Biotechnology. After washing, slides were incubated with secondary antibody conjugated to Alexa Fluor dye (Invitrogen) at room temperature for an hour in the dark, washed again, stained with 4′,6-diamidino-2-phenylindole (DAPI), and mounted with Prolong Gold Antifade (Invitrogen, Grand Island, NY, USA) to visualize the nuclei. Images were captured on a confocal microscope (LSM 780; Zeiss, Jena, Germany).

### RNA interference

**S**B2-4 and SB7 cell lines were transiently knocked down with a validated siRNA targeting YAP (Ambion, #s76159): 5′-ttaaatcacaacgatcaga-3′. Cells grown in 96-well plates were transfected with 20 nM siRNA using Lipofectamine RNAiMAX (Life Technologies) (25% of the manufacturer’s recommended concentration was used to minimize cell toxicity observed at higher concentrations). Control sequences provided by manufacturer were transfected in parallel. Cells were lysed 72 h following transfection, and immunoblot analysis was performed.

### *In vitro* cell death studies

To evaluate cytotoxicity, cells were grown in 96-well plates (5 × 10^3^ cells per well) and respective treatments were added after 24 hours. Cells were assayed for death using the CellTiter-Blue assay (CellTiter-Blue Cell Viability Assay, Promega) performed according to the manufacturer’s instructions.

### Statistics

Data represent at least three independent experiments and are expressed as mean ± SEM. Differences in experiments with two groups were compared using the two-tailed Student *t*-test or the Fisher’s exact test. Differences were considered as significant at levels of *p* < 0.05.

## SUPPLEMENTARY MATERIALS FIGURES



## Abbreviations

α-SMA: alpha-smooth muscle actin
CCA: cholangiocarcinoma
CIN: chromosomal instability
CK: cytokeratin
CNV: copy number variation
FGFR: fibroblast growth factor receptor
FOXM1: forkhead box M1
HCC: hepatocellular carcinoma
HNF4α: hepatocyte nuclear factor 4 alpha
huYAP: human YAP
IL-33: interleukin 33
muAkt: murine Akt
MPseq: mate-pair sequencing
PTEN: phosphatase and tensin homolog
siRNA: small interfering RNA
SOX9: SRY (Sex Determining Region Y)-Box 9
TCGA: the cancer genome atlas
TEAD4: TEA domain transcription factor 4
YAP: yes-associated protein
