# Supplementary material for: YAP-associated chromosomal instability and cholangiocarcinoma in mice
Source: Oncotarget. 2017 Dec 22;9(5):5892–905. doi: 10.18632/oncotarget.23638 (PMC5814182; doi:10.18632/oncotarget.23638)
Supplement: Supplementary file 1 [file oncotarget-09-5892-s001.pdf]

YAP-associated chromosomal instability and cholangiocarcinoma in mice

SUPPLEMENTARY MATERIALS

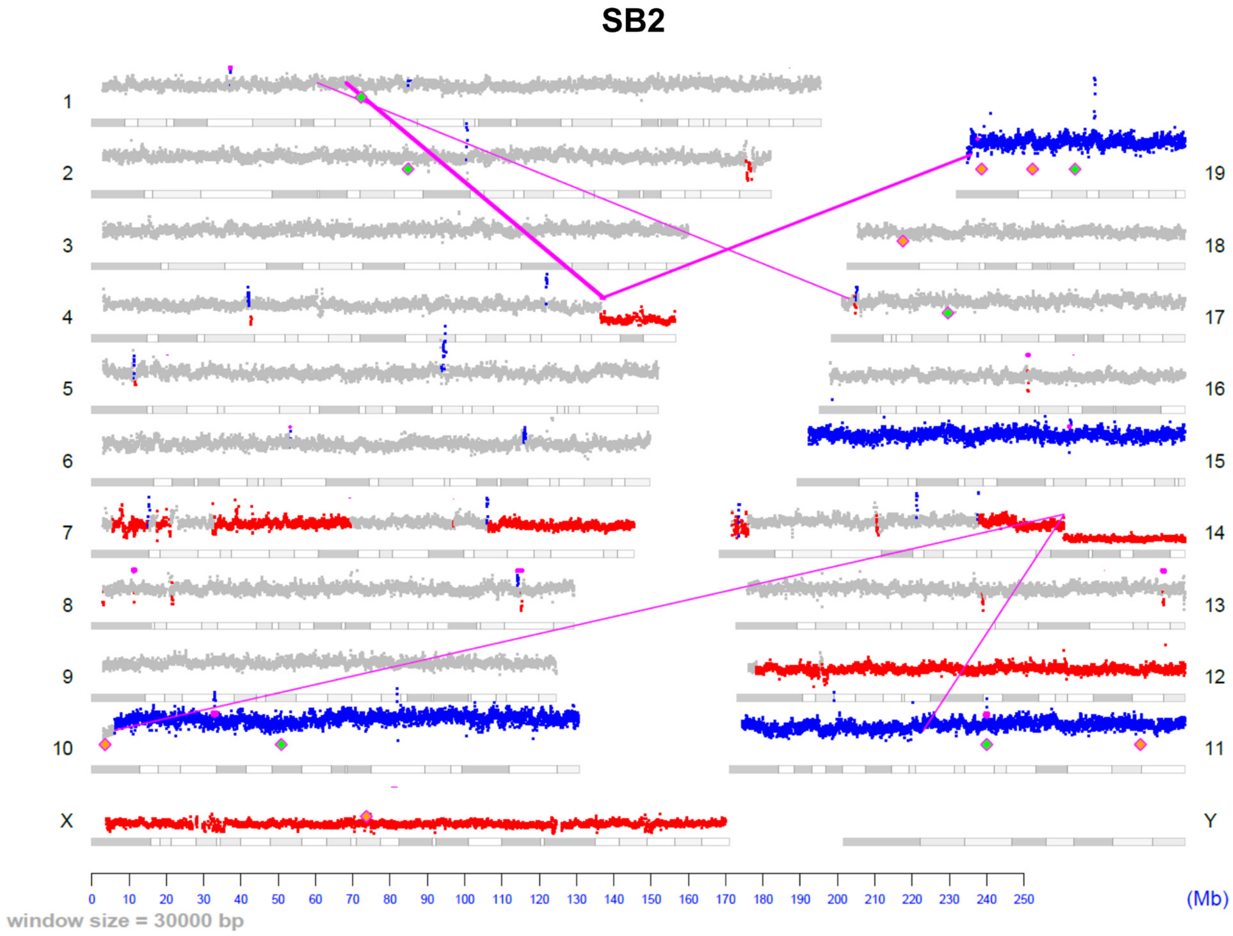

Supplementary Figure 1: Genome plot for cell line SB2. The genome plot presents the full mate-pair analysis for cell line SB2. Autosomes are numbered 1 to 19 and allosomes are labeled X and Y. Each chromosome panel is represented by its (a) G banding phenotype, (b) dots denoting sequencing coverage and (c) diamonds representing integration sites of huYAP (*orange diamonds*) or muAkt (*green diamonds*). Each dot represents 30000 bases. Regions calculated to have normal copy number level are colored gray. Regions of data are colored blue for gain of genetic material or red for loss of genetic material. Inter- and intra-chromosomal breakpoint junctions are presented as magenta lines linking two breakpoint positions.

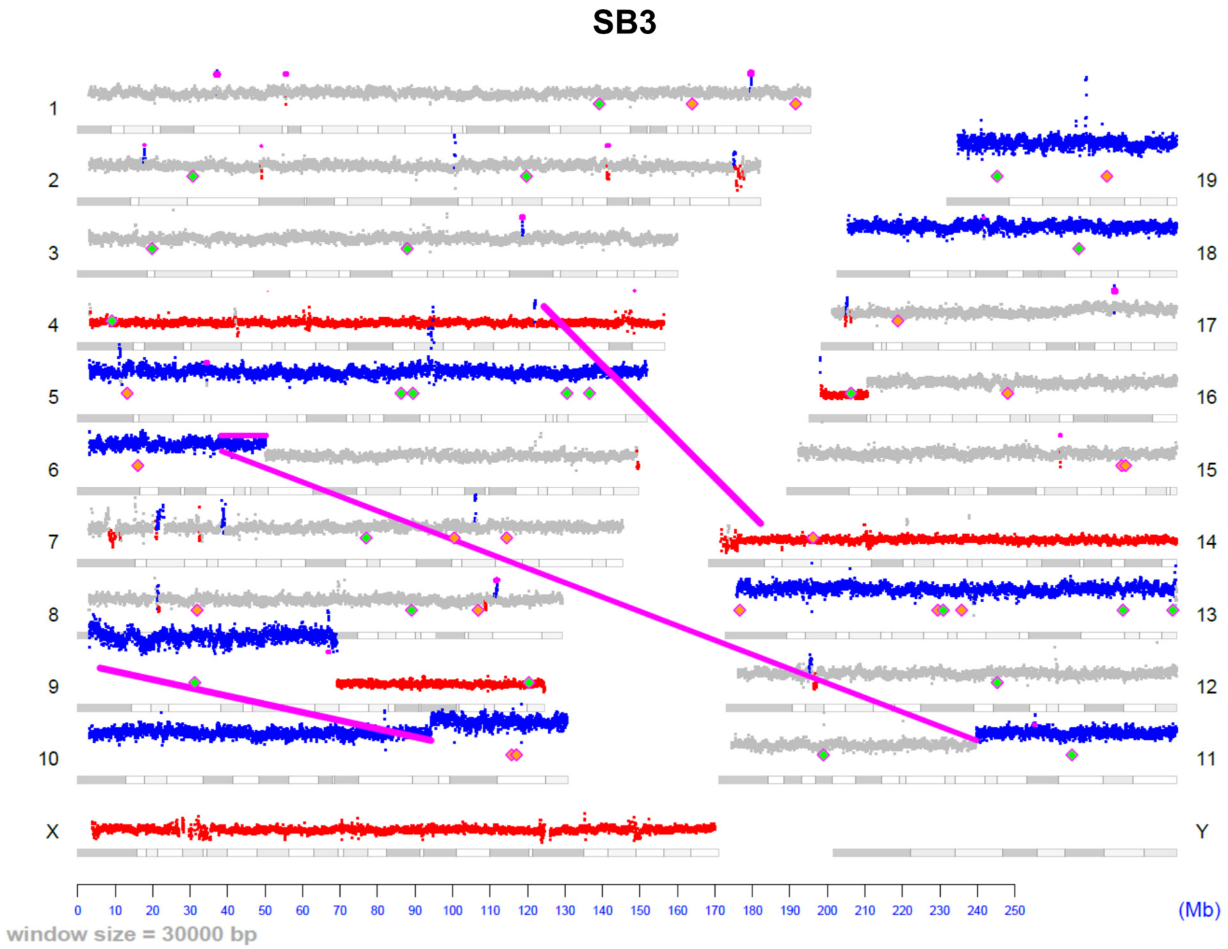

Supplementary Figure 2: Genome plot for cell line SB3. The genome plot presents the full mate-pair analysis for cell line SB3. Autosomes are numbered 1 to 19 and allosomes are labeled X and Y. Each chromosome panel is represented by its (a) G banding phenotype, (b) dots denoting sequencing coverage and (c) diamonds representing integration sites of huYAP (*orange diamonds*) or muAkt (*green diamonds*). Each dot represents 30000 bases. Regions calculated to have normal copy number level are colored gray. Regions of data are colored blue for gain of genetic material or red for loss of genetic material. Inter- and intra-chromosomal breakpoint junctions are presented as magenta lines linking two breakpoint positions.

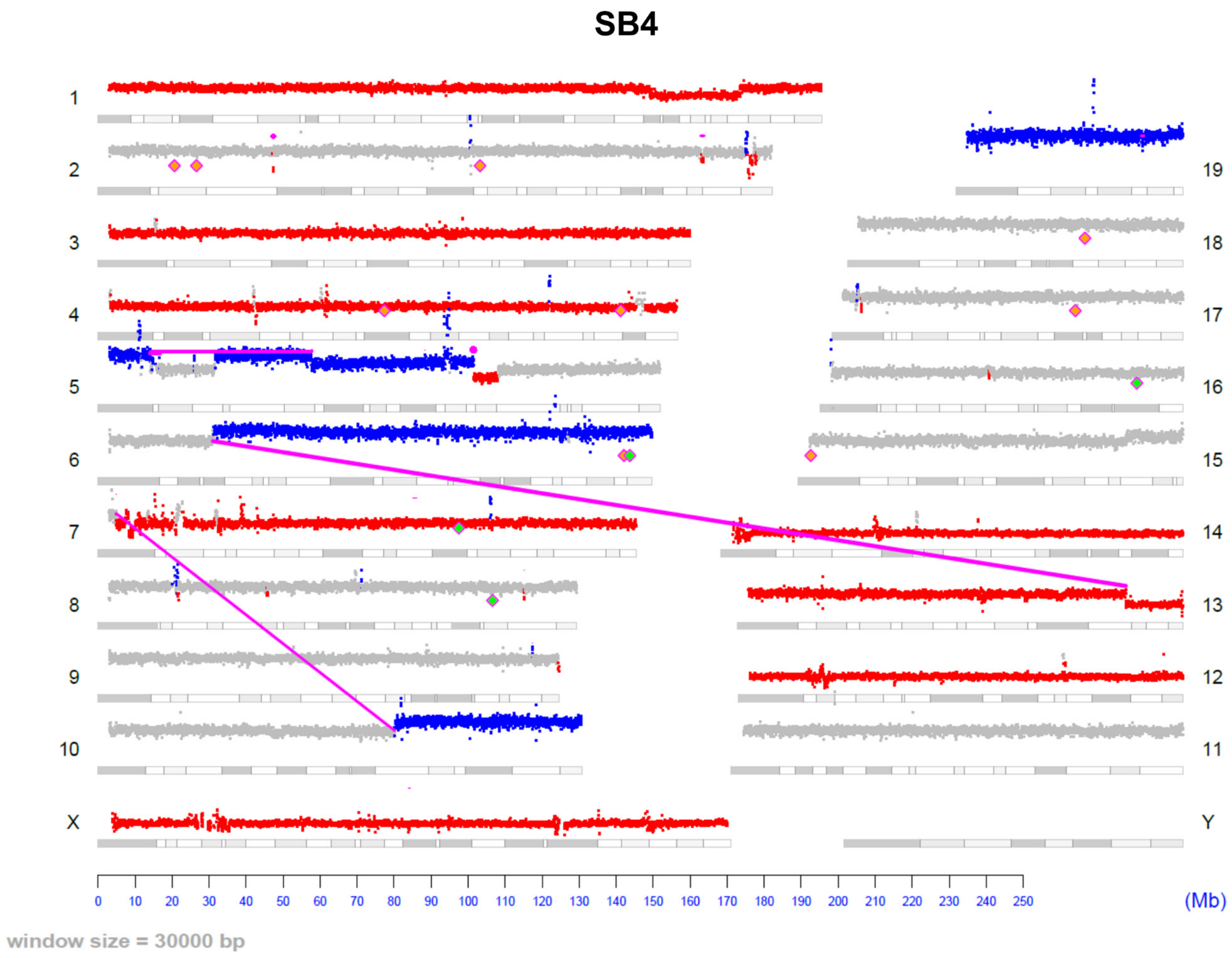

Supplementary Figure 3: Genome plot for cell line SB4. The genome plot presents the full mate-pair analysis for cell line SB4. Autosomes are numbered 1 to 19 and allosomes are labeled X and Y. Each chromosome panel is represented by its (a) G banding phenotype, (b) dots denoting sequencing coverage and (c) diamonds representing integration sites of huYAP (*orange diamonds*) or muAkt (*green diamonds*). Each dot represents 30000 bases. Regions calculated to have normal copy number level are colored gray. Regions of data are colored blue for gain of genetic material or red for loss of genetic material. Inter- and intra-chromosomal breakpoint junctions are presented as magenta lines linking two breakpoint positions.

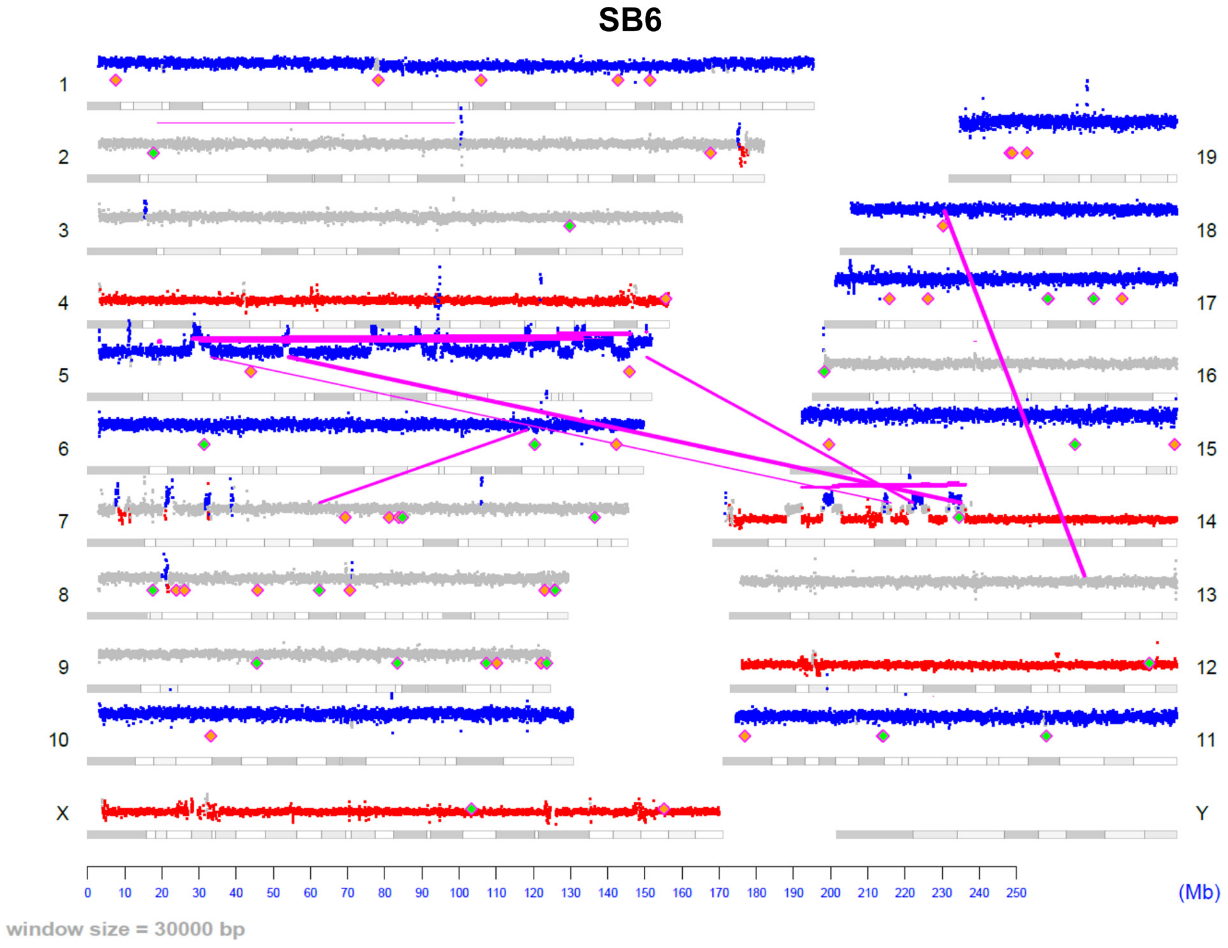

Supplementary Figure 4: Genome plot for cell line SB6. The genome plot presents the full mate-pair analysis for cell line SB6. Autosomes are numbered 1 to 19 and allosomes are labeled X and Y. Each chromosome panel is represented by its (a) G banding phenotype, (b) dots denoting sequencing coverage and (c) diamonds representing integration sites of huYAP (*orange diamonds*) or muAkt (*green diamonds*). Each dot represents 30000 bases. Regions calculated to have normal copy number level are colored gray. Regions of data are colored blue for gain of genetic material or red for loss of genetic material. Inter- and intra-chromosomal breakpoint junctions are presented as magenta lines linking two breakpoint positions.

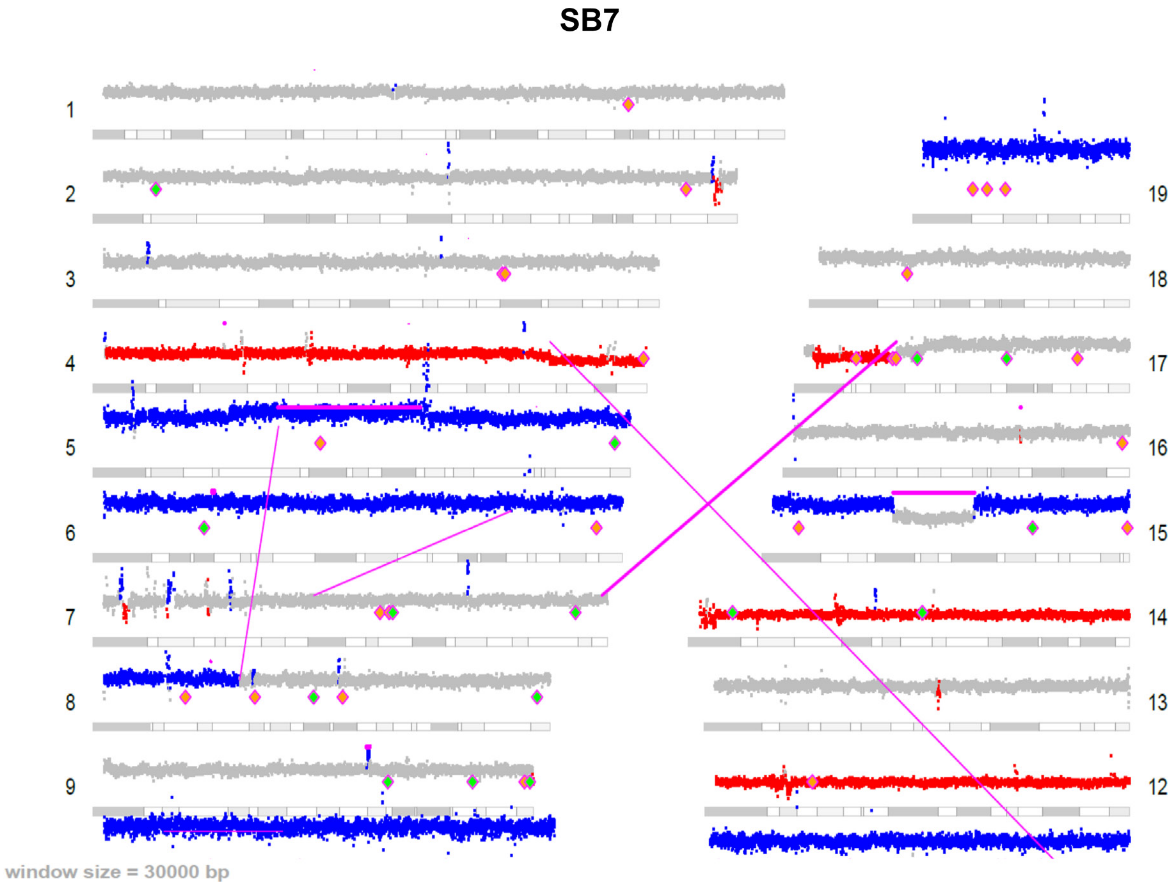

Supplementary Figure 5: Genome plot for cell line SB7. The genome plot presents the full mate-pair analysis for cell line SB7. Autosomes are numbered 1 to 19 and allosomes are labeled X and Y. Each chromosome panel is represented by its (a) G banding phenotype, (b) dots denoting sequencing coverage and (c) diamonds representing integration sites of huYAP (*orange diamonds*) or muAkt (*green diamonds*). Each dot represents 30000 bases. Regions calculated to have normal copy number level are colored gray. Regions of data are colored blue for gain of genetic material or red for loss of genetic material. Inter- and intra-chromosomal breakpoint junctions are presented as magenta lines linking two breakpoint positions.

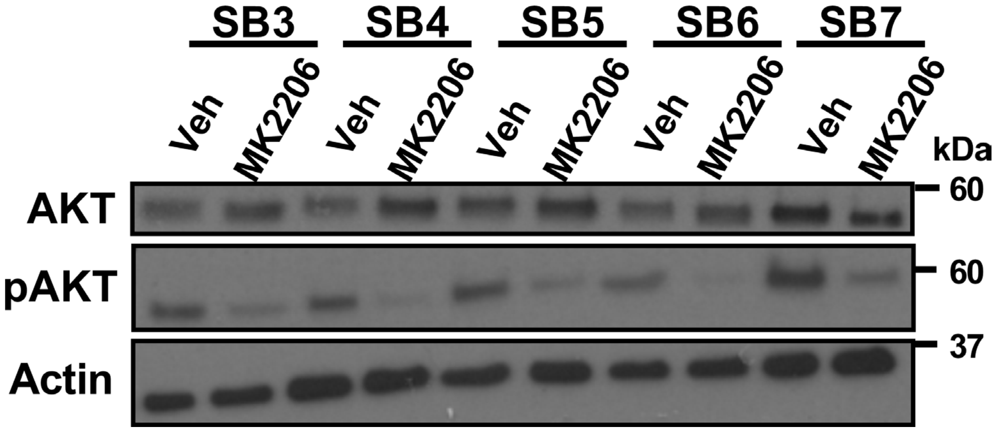

Supplementary Figure 6: MK2206 inhibits AKT activating phosphorylation. SB3-7 cells were treated with vehicle (veh) or MK2206 (1 μM) for 48 h. Whole cell lysates were subsequently prepared and subjected to immunoblot analysis of AKT and phospho-AKT. β-actin was used as a loading control.
